# Bridgmanite’s ferric iron content determined Earth’s oxidation state

**DOI:** 10.1038/s41561-025-01725-0

**Published:** 2025-06-23

**Authors:** Fei Wang, Lin Wang, Hongzhan Fei, Nobuyoshi Miyajima, Catherine McCammon, Daniel J. Frost, Tomoo Katsura

**Affiliations:** 1 Bayerisches Geoinstitut, University of Bayreuth, Bayreuth, Germany; 2 School of Earth Sciences, Zhejiang University, Hangzhou, China

**Keywords:** Mineralogy, Geochemistry, Petrology

## Abstract

Bridgmanite, a magnesium-rich silicate perovskite, is the most prevalent mineral in Earth’s lower mantle and contains substantial quantities of ferric (oxidized) iron, even in equilibrium with iron metal. Mixing of oxygen-rich material from the lower mantle could have raised the oxidation state of the upper mantle to its present level after the more reducing conditions during core formation. However, it remains unclear how the lower-mantle oxygen content was established to achieve this level. Here we use high-pressure and temperature multi-anvil experiments at known oxygen fugacities to show that the bridgmanite ferric iron content is independent of pressure but decreases with temperature. Using these data, we build a thermodynamic model to calculate the ferric iron content of the lower mantle as bridgmanite crystallized from a reduced magma ocean in the early Earth. We determine that this ferric iron content would have been sufficient to explain the current upper mantle’s ferric iron content after whole mantle mixing.

## Abstract (web summary)

As bridgmanite crystallized from a reduced magma ocean, it left an oxidized lower mantle that may explain the oxidation state of the current upper mantle after mixing, according to high-pressure and high-temperature multi-anvil experiments.

## Main

During Earth’s accretion, relatively reducing redox conditions prevailed in the silicate mantle due to equilibrium with core-forming iron-rich liquid metal^1,2^. However, the current upper mantle is more oxidized than if it were in equilibrium with metallic iron, which is reflected in its higher ferric iron content and the dominance of H_2_O and CO_2_ in mantle-derived volcanic gases^3–5^. This implies that the oxidation state of the upper mantle has increased since core formation^6,7^. Currently, however, the cause remains uncertain.

One possible explanation is based on the preference of bridgmanite in the lower mantle for ferric iron, which results from a charge-coupled substitution of Fe^3+^ and Al^3+^ cations at the two sites of the bridgmanite structure^8,9^. This substitution is favourable even at redox conditions where metallic iron is stable, which means that when bridgmanite crystallized from an initially reduced magma ocean, its preference for ferric iron would have driven a process of charge disproportionation, that is, 3 FeO = Fe + Fe_2_O_3_, creating metallic iron^8^. The separation of this metallic iron to the core would have raised the bulk oxygen content of the remaining lower mantle and the oxidation state of the entire mantle after whole mantle mixing.

Aspects of this scenario remain unclear, however, mainly because the Fe^3+^/ΣFe ratio (Σ denotes summation) of bridgmanite has been determined at well-defined oxygen fugacities only at conditions compatible with the topmost lower mantle. At these conditions, bridgmanite can have a Fe^3+^/ΣFe ratio larger than 0.5 in equilibrium with iron metal^8,9^. If this level of ferric iron was produced by charge disproportionation throughout the entire lower mantle and the resulting metal separated to the core, the lower mantle would have been too oxidized to have substantially mixed with the upper mantle^8,10^. Partial loss of disproportionated metallic iron from the lower mantle, on the other hand, is difficult to explain^11^, as is partial mixing between the upper and lower mantle, given that the upper mantle oxidation state has remained essentially constant during the geological record^12^. A further complicating aspect is that recent studies have argued that charge disproportionation of FeO may have occurred within the silicate melt at lower-mantle depths^13–17^. Even in this case, however, the lower-mantle oxidation state would have still been fixed at the point of bridgmanite crystallization, whereas contemporaneous loss of disproportionated metallic iron could have still left a lower mantle too oxidized to have mixed substantially with the upper mantle.

To clarify the oxidation state of the lower mantle when it solidified and its potential role in raising the redox state of the entire mantle, we have determined the Fe^3+^/ΣFe ratios of two bridgmanite compositions, Mg_0.91_Fe_0.10_Al_0.08_Si_0.91_O_3_ and Mg_0.88_Fe_0.11_Al_0.13_Si_0.88_O_3_, hereafter referred to as Fe10Al8 (lower Al/Fe ratio) and Fe11Al13 (higher Al/Fe ratio), coexisting with ferropericlase and an oxygen fugacity sensor (Fe-Ir alloy), as a function of pressure to mid-lower-mantle conditions. The results are combined with data previously obtained as a function of oxygen fugacity and temperature^9,18^ to build a complete thermodynamic model that describes how the Fe^3+^/ΣFe ratio in bridgmanite changed with depth throughout the lower mantle at different times in Earth’s history. Our model indicates that the bridgmanite that crystallized from a global magma ocean had a lower ferric iron content than previously believed, and this content can fully explain the current ferric iron content in the upper mantle after whole mantle mixing.

## Phase assemblages and element compositions

**Fig. 1:**
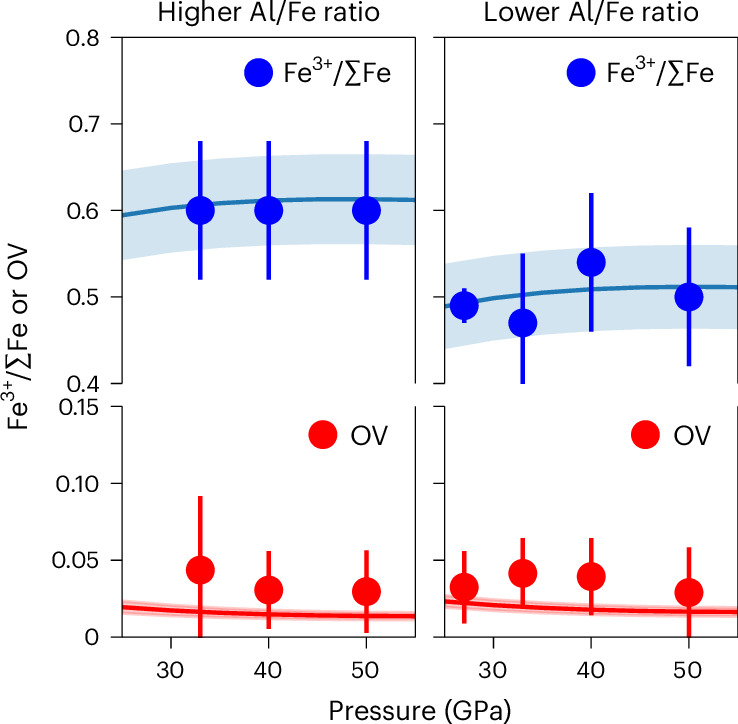
Fe^3+^/ΣFe ratio and oxygen vacancy (OV) component as a function of pressure in two different Al- or Fe-bearing bridgmanite compositions. Data are presented as experimental values ± 1σ (standard deviation). The solid lines are from our thermodynamic model using 2,300 K and oxygen fugacity ΔIW = 1.5. The shaded region is the uncertainty when the oxygen fugacity fluctuation is 0.5 log unit and temperature fluctuation is 50 K.

Multi-anvil experiments were performed between 27 and 50 GPa at 2,300 K. The recovered samples were comprised of assemblages of bridgmanite, ferropericlase and Ir-Fe alloy (Supplementary Table 1 and Supplementary Figs. 1 and 2). Oxygen fugacities, determined from the ferropericlase FeO content and the coexisting Ir-Fe alloy’s Fe content^19^, were relatively constant throughout the samples with a mean value of 1.4 ± 0.5 log units above the iron-wüstite buffer (ΔIW). The Fe^3+^/ΣFe ratios of the recovered samples from Fe10Al8 and Fe11Al13 were determined using Mössbauer spectroscopy to be 0.5 and 0.6, respectively, throughout the entire pressure range investigated (Supplementary Fig. 3). The dependence of the bridgmanite Fe^3+^/ΣFe ratio on the Al content when other parameters, such as oxygen fugacity, stay similar is in agreement with previous studies^8,9^. For both compositions, there was no detectable change in the Fe^3+^/ΣFe ratios with increasing pressure within the uncertainties, as shown in Supplementary Table 2 and Fig. 1.

## Fe^3+^ and Al substitutions in bridgmanite

Bridgmanite has the general formula ABO_3_. Trivalent cations, predominantly Al^3+^ and Fe^3+^, can substitute onto the A and B sites through two competing mechanisms^9,18,20–24^. One is a charge-coupled (CC) substitution mechanism, where two trivalent cations simultaneously substitute onto A and B sites. The other is an oxygen vacancy (OV) substitution mechanism, where two trivalent cations substitute onto B sites, with charge balance occurring by the formation of an oxygen vacancy. The proportions of the OV and CC components were determined from the experimentally measured proportions of Si^4+^, Mg^2+^, Fe^2+^, Fe^3+^ and Al^3+^ (refs. ^9,18,20–23^). Figure 1 shows there is a slight decrease of the OV fraction with increasing pressure. The decreasing trend agrees with previous studies^22–24^. Our result differs from those from systems that contain only Al^3+^ as the trivalent element^23^ and from basaltic systems^24^, where the OV component was observed to rapidly decrease with pressure and virtually disappeared at 40 GPa and 50 GPa, respectively. The formation of the OV component is enhanced by increasing temperature^18,21^ and the temperature in our experiments was 300 K higher than in those experiments^23,24^. The observation that the OV component was still present in our experiments at 50 GPa indicates that although it is suppressed by pressure, the OV component is still probably present in bridgmanite at typical geotherm conditions^25^ to mid-lower-mantle depths. Such a component may have important effects on the transport properties of the lower mantle^20–23^.

## Thermodynamic model

We derive a thermodynamic model to interpolate the composition of bridgmanite over the range of conditions and compositions encountered in this and two previous well-characterized studies^9,18^. The complete dataset covers wide ranges of temperature, 1,800 K‒2,600 K, oxygen fugacity, ΔIW −1 to +8, pressure, 25 GPa‒50 GPa and various bridgmanite Al and Fe contents. We describe the variation in bridgmanite (Bdm) composition using six endmembers, MgSiO_3_, MgAlO_2.5_, FeSiO_3_, FeFeO_3_, AlAlO_3_ and FeAlO_3_, and coexisting ferropericlase (Fp) with two endmembers, MgO and FeO, neglecting the small Fe_2_O_3_ component. The site occupancies in the two phases at equilibrium are calculated by considering four independent equilibria

2.1 $${2{\rm{MgAlO}}}_{2.5}\left({\rm{Bdm}}\right)={{\rm{AlAlO}}}_{3}({\rm{Bdm}})+2{\rm{MgO}}({\rm{Fp}})$$

2.2 $${{\rm{MgSiO}}}_{3}\left({\rm{Bdm}}\right)+{\rm{FeO}}\left({\rm{Fp}}\right)={{\rm{FeSiO}}}_{3}\left({\rm{Bdm}}\right)+{\rm{MgO}}\left({\rm{Fp}}\right)$$

2.3 $$2{\rm{FeO}}\left({\rm{Fp}}\right)+\frac{1}{2}{\rm{O}}_{2}={{\rm{FeFeO}}}_{3}\left({\rm{Bdm}}\right)$$

2.4 $${{\rm{FeFeO}}}_{3}\left({\rm{Bdm}}\right)+{{\rm{AlAlO}}}_{3}\left({\rm{Bdm}}\right)={2{\rm{FeAlO}}}_{3}\left({\rm{Bdm}}\right)$$

The thermodynamic parameters of each endmember in each phase specified above and their interaction parameters were optimized by minimizing the sum of the squared Gibbs free energy changes over equations (2.1)–(2.4) (Methods). Volume changes and, therefore, the pressure dependences of these equilibria are constrained using recently measured volume and equation of state data for the specific endmembers^26,27^. The calculated bridgmanite Fe^3+^/ΣFe ratios and OV fractions are shown to be in good agreement with the original experimental data in Supplementary Figs. 4 and 5.

## Pressure effect on iron oxidation in bridgmanite

**Fig. 2:**
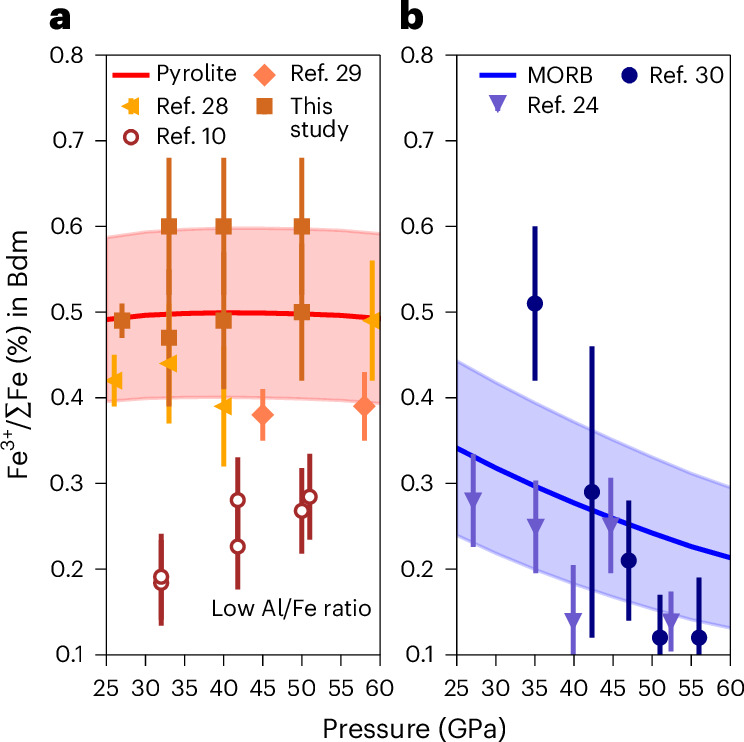
Ferric iron ratio in bridgmanite as a function of pressure showing how our model reproduces experimental results and literature data. Data denoted as from this work are experimental values ± 1σ. Other data are the values and error bars reported in the cited references and are usually also experimental values ± 1σ. **a**,**b**, The red and blue lines are from our model for bridgmanite that coexists with ferropericlase (**a**) and stishovite (**b**). The temperatures are 2,300 K and 2,000 K for the pyrolite and mid-ocean ridge basalt (MORB) system, respectively, both with an oxygen fugacity of IW, with the shaded region extending from ΔIW −1.0 to +1.0.

We further evaluate our result and model by comparing it with the results from other laser-heated diamond anvil cell studies where the oxygen fugacity was not determined but can be assumed to have remained relatively constant. In Fig. 2a we calculate the Fe^3+^/ΣFe ratio of bridgmanite within a pyrolite bulk composition as a function of pressure at 2,300 K and an oxygen fugacity of IW, with the shaded region extending from ΔIW −1.0 to +1.0. A comparison with the results of Prescher et al.^28^ and Piet et al.^29^ who performed experiments with a similar composition and temperature shows good agreement over this pressure range and reveals plausible oxygen fugacities between IW and ΔIW −1.0.

To further verify that the model has captured the thermodynamic properties of bridgmanite, we also extend the comparison to experiments performed using more silica-rich bulk compositions such as basalt. In this case, reactions (2.1), (2.2) and (2.3) that involve ferropericlase do not occur but are replaced by equilibria involving stishovite,

2.5 $${3{\rm{FeSiO}}}_{3}\left({\rm{Bdm}}\right)+{{\rm{AlAlO}}}_{3}\left({\rm{Bdm}}\right)={3{\rm{SiO}}}_{2}\left({\rm{Sti}}\right)+{2{\rm{FeAlO}}}_{3}\left({\rm{Bdm}}\right)+{\rm{Fe}}$$

2.6 $${2{\rm{MgAlO}}}_{2.5}\left({\rm{Bdm}}\right)+{2{\rm{SiO}}}_{2}\left({\rm{Sti}}\right)={2{\rm{MgSiO}}}_{3}\left({\rm{Bdm}}\right)+{{\rm{AlAlO}}}_{3}\left({\rm{Bdm}}\right)$$

Equilibrium (2.5) has a positive volume change at upper lower-mantle conditions^9^, which implies that in contrast to pyrolite compositions, pressure should favour decreasing ferric iron in bridgmanite for stishovite-bearing compositions such as basalt. To quantitatively examine this effect, in Fig. 2b we show calculated the bridgmanite Fe^3+^/ΣFe ratio for a mid ocean ridge basalt composition at the same oxygen fugacity as in Fig. 2a and 2,000 K. Our model indicates that the bridgmanite Fe^3+^/ΣFe ratio indeed decreases as expected, from 34% to 22% between 25 and 60 GPa. The model is in good agreement with the results of Ishii et al.^24^ for a basaltic composition, and the decreasing trend with increasing pressure is reproduced. Data from Shim et al.^30^ are also shown, but in this previous study, a SiO_2_ phase is only reported to be present below 40 GPa.

Supplementary Fig. 4 shows a 1:1 comparison with literature bridgmanite Fe^3+^/ΣFe ratios in experiments over wide ranges of pressure (27‒110 GPa), temperature (1,800‒26,00 K), Al/Fe ratio (0‒1.3) and oxygen fugacity (ΔIW −2 to +8). The good agreement with our thermodynamic model indicates that previous discrepancies regarding the pressure effect actually result from other variables, such as differing oxygen fugacities, imposed silica activities or low Al/Fe ratios that were not previously considered. For example, the data from Andrault et al.^10^ appear to be in poor agreement with our study (Fig. 2a), however, this results from their bulk composition containing twice as much iron compared to pyrolite. Our model is able to reproduce experimental data covering wide ranges of oxygen fugacity, composition, pressure and temperature, with the pressure dependence also consistent with the now well-determined molar volumes of bridgmanite endmembers^26,27^.

## Deep Earth’s oxygen reservoir

As the Earth formed, upper mantle material would have been in equilibrium with core-forming iron-rich metal, which would have left it with very low levels of ferric iron. However, the current upper mantle has a Fe^3+^/ΣFe ratio in the range 0.02‒0.06^3–5^. One explanation for this is that the upper mantle ferric iron content was raised through mixing with oxygen-rich material from the lower mantle. The lower mantle would have been oxygen rich due to the ferrous iron charge disproportionation that occurred as bridgmanite formed and the separation of the resulting iron metal to the core^8^.

A problem arises, however, because partial separation of this metal to the core is hard to explain^11^, but if all the metal separated, then based on previous estimates of 0.5‒0.6 for the bridgmanite Fe^3+^/ΣFe ratio^9^, then whole mantle mixing would result in the ferric iron content of the upper mantle being 3‒5 times higher than its current level^11^. If no metal separated, on the other hand, then the lower mantle would not have increased in its oxygen content and would not be able to oxidize the upper mantle through mixing.

This problem is mainly caused by an incomplete picture of the bridgmanite ferric iron content in the lower mantle as it crystallized from a magma ocean. There is evidence that bridgmanite formed from silicate melt at the top of the lower mantle in equilibrium with iron metal would have a lower Fe^3+^/ΣFe ratio of 0.14‒0.25 compared to the value of 0.5‒0.6 at modern day geotherm conditions^8,31^. As our results show that pressure has no effect on the Fe^3+^/ΣFe ratio in bridgmanite in pyrolytic systems, this lower level of ferric iron content would have been maintained along the lower-mantle solidus and along the boundary marking the first crystallization of bridgmanite from the magma ocean.

**Fig. 3:**
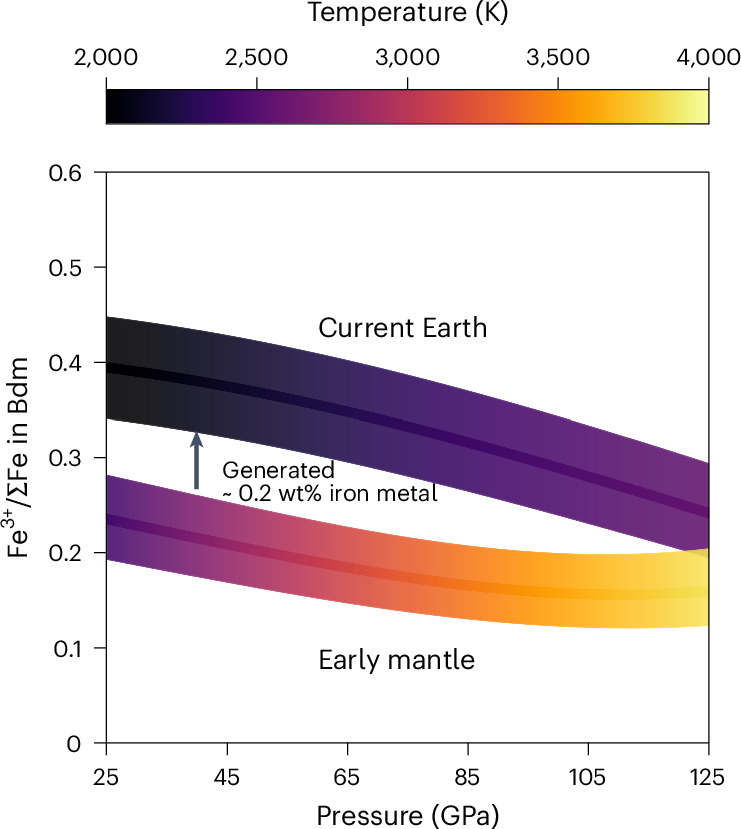
Calculated Fe^3+^/ΣFe ratio in bridgmanite in the lower mantle with an oxygen fugacity of IW −1 along the current mantle geotherm compared with that along an early Earth’s lower-mantle solidus, that is, the average of the solidi in the literature^33–36^ and a mantle oxygen fugacity of IW −2. The colour indicates the temperature. The combined effect of temperature, pressure and oxygen fugacity determines the Fe^3+^/ΣFe ratio in bridgmanite. The shaded area indicates the uncertainty calculated using error propagation, propagated from temperature and oxygen fugacity uncertainties of 200 K and half a log unit, respectively. To charge balance the increased ferric iron in bridgmanite resulting from ferrous iron disproportionation as the Earth cooled, approximately 0.2 wt% iron metal would have been generated.

To show this quantitatively, we use our model combined with a low-spin ferropericlase FeO component from Stixrude and Lithgow–Bertelloni^32^ to calculate the Fe^3+^/ΣFe ratio in bridgmanite along a mantle solidus determined by taking an average over literature solidi^33–36^ (Fig. 3). The oxygen fugacity was set to IW −2.0(5), to be compatible with that in the magma ocean era and with the iron oxide content of the mantle^1,17^. The calculated average Fe^3+^/ΣFe ratio in bridgmanite is 0.17(3), which gives a lower mantle that has an overall Fe^3+^/ΣFe ratio of 0.09(2). Because the lower mantle comprises 75 wt% of the mantle, after homogenization with a near Fe^3+^-free upper mantle, the whole mantle will have an Fe^3+^/ΣFe ratio of 0.07(2). This is an upper limit set by the nominal case that all the iron generated by charge disproportionation above the solidus separated to the core. This agrees with the estimates for the upper mantle of 0.02‒0.06^3–5^.

The lower-mantle solidus will define the upper limit of the lower-mantle oxygen content, because disproportionated metal would not have been able to separate to the core at sub-solidus conditions. Several physical processes would have facilitated the loss to the core of liquid iron produced through disproportionation in bridgmanite. First, based on thermodynamic models, the extent of bridgmanite crystallization would not change substantially with depth as the adiabat of a crystallizing magma ocean mirrors the solidus in the lower mantle^37,38^ so that descending metal liquid would not have encountered underlying sub-solidus mantle. Second, liquid iron appears to wet mineral grain boundaries at high pressures and temperatures and should, therefore, be able to percolate through sub-solidus silicate assemblages^39^, potentially aided by high stresses caused by rapid convection^40^ or accumulation into diapirs^41^. Although there are substantial uncertainties, experimental studies place the iron metal solidus very close to the lower-mantle solidus (Supplementary Fig. 6), so it is plausible that the crystallization of solid iron ultimately stopped its loss to the core. Finally, as the magma ocean crystallized, it is proposed to have reached iron-sulfide saturation. The separation of this sulfide liquid to the core, as the so-called Hadean matte, is required to explain the abundance of highly siderophile elements in the mantle^42^. Disproportionated liquid iron metal may have been removed through alloying with this late-stage separating sulfide melt.

Although our model cannot be extended into the region of silicate melting, based on the temperature dependence of the bridgmanite Fe^3+^/ΣFe ratio, the value at crystallization will be close to, but probably lower than, the solidus value. The fact that the bridgmanite Fe^3+^/ΣFe ratio, integrated over the entire lower-mantle solidus, is so close to the ratio of the mantle as a whole after mixing, provides good support for the role of the lower mantle in mantle oxidation after core formation. It has been proposed that changes in lower-mantle redox state could result in density differences that would make oxidized regions more buoyant^43,44^. However, as demonstrated in our study, bridgmanite crystallized from a magma ocean would have a very narrow range of Fe^3+^/ΣFe ratio throughout the whole of the lower mantle that is unlikely to influence mantle convection (Fig. 3).

Before bridgmanite crystallized from the magma ocean, it is likely that some ferrous iron disproportionation already occurred within the silicate melt, with loss of the resulting metal to the core leaving the magma enriched in ferric iron^13–17^. However, recent high-pressure measurements indicate that the level of ferric iron produced in the magma would have been less than that produced as bridgmanite crystallizes^16^. Therefore, as bridgmanite crystallized, further disproportionation would have had to occur, which would ultimately fix the ferric iron content of the solid lower mantle. It is possible that some iron liquid may have been trapped in the mantle at near-solidus conditions. Further studies of metal separation would be needed to clarify this. Therefore, the ferric iron content in the mantle at the solidus provides the upper-limiting endmember for the amount of ferric iron in the lower mantle, whereas that produced by disproportionation of FeO in the liquid magma provides the lower limit.

The calculated lower bridgmanite Fe^3+^/ΣFe ratios, and the present proposal that loss of iron metal to the core occurred upon bridgmanite crystallization, does not contradict the argument that the current lower mantle is iron metal saturated. This is because, as the mantle cooled below the solidus, iron would have been generated to charge balance the increasing Fe^3+^/ΣFe ratio of bridgmanite with decreasing temperature^18^. We estimate that approximately 0.2 wt% iron metal has been generated at the top of lower mantle through ferrous iron charge disproportionation as the lower mantle cooled from the solidus to its present level. For this estimate, we used the Fe^3+^/ΣFe ratio in bridgmanite calculated along a current mantle geotherm^25^ for a pyrolite composition with an oxygen fugacity of IW −1, shown in Fig. 3. This estimate for the current lower-mantle^9,18^ oxygen fugacity is slightly higher than at bridgmanite crystallization due to the decrease in temperature, which raises the iron concentration in ferropericlase and increases in the nickel content of the iron metal alloy. Figure 3 shows that the present ratios are higher than those in the early Earth, which is mainly due to the lower temperatures. In addition, whereas in the upper lower mantle, iron is distributed almost equally between bridgmanite and ferropericlase, in the deeper mantle, due to a Fe^2+^ spin transition in ferropericlase, iron is partitioned out of the coexisting bridgmanite^10,32^. Using the available data^10,29^, we estimate that the Fe^3+^/ΣFe ratio at the base of the lower mantle is just over half the value at the top of the lower mantle.

## Methods

### Starting materials

We prepared two starting materials A and B using the compositions shown in Supplementary Table 6. Starting material A contained less Al (lower Al/Fe ratio) than starting material B (higher Al/Fe ratio). The reagent grades of the oxide powders were mixed by finely grinding them in an agate mortar. Each of the mixtures was melted by heating to 2,000 K at room pressure and then quenched in water to produce a glass. A powder of each glass was cold pressed into a pellet and placed in a CO-CO_2_ gas mixing furnace at oxygen fugacity 2 log unit below the fayalite-magnetite-quartz (FMQ) buffer for 48 h at 1,423 K to reduce the ferric iron to ferrous. 5 wt% (Mg_0.9_Fe_0.1_)O ferropericlase without isotopic enrichment and 5 wt% Ir metal, which became an Ir-Fe alloy during the experiment and was used as an oxygen fugacity sensor, were then added to each mixture.

### High-pressure and high-temperature experiments

High-pressure and high-temperature experiments were conducted at 27–50 GPa and 2,300 K for 24–40 h using a 15 MN Osugi-type multi-anvil high-pressure apparatus (IRIS-15) at the Bayerisches Geoinstitut (BGI)^45,46^. The long duration of ≥ 24 h at 2,300 K ensured equilibrium in the samples because experiments containing similar phases have been shown to reach equilibrium when performed at a lower temperature and for a shorter period of time^24,47^. Depending on the experimental pressure, the starting material was loaded into a capsule made of platinum foil with an outer diameter of either 1.0 mm (27 GPa) or 0.4 mm (>27 GPa). For the 27 GPa experiments, a standard BGI 7/3 cell assembly with a LaCrO_3_ furnace was used with TF05-grade tungsten carbide anvils. For the experiments at pressures higher than 27 GPa, a user-designed 5.7/1.5 cell was used (Supplementary Fig. 7). This used TF05-grade tungsten carbide anvils for 33 and 40 GPa or TJS01-grade tungsten carbide anvils with 1.5 mm truncation and 1.0° tapering for 40 and 50 GPa.

The sample was slowly compressed to the desired pressure over 5‒6 h. Then the temperature was increased at a rate of 100 K per min to 2,300 K. The temperature was measured using a W97Re3–W75Re25 (D type) thermocouple, which was used without applying a pressure correction to the electromotive force. After heating, the sample was quenched by turning off the power. The sample was decompressed to room pressure over 18‒24 h at room temperature.

### Sample characterization

The recovered samples were embedded in epoxy resin and making a cross section for textual observation using backscattered electron images obtained with a ZEISS Gemini 1530 scanning electron microscope. Quantitative chemical analyses were obtained using a JEOL JXA-8200 electron microprobe analyser operated with a point beam (~1 μm), an acceleration voltage of 15 kV, a beam current of 5 nA and counting time of 10 s. The calibration standards were single crystal enstatite for Mg and Si, corundum for Al, Ir metal and Fe metal. Because some iron loss occurred to the platinum capsule, grains within 10 μm of the capsule wall were excluded from the analyses. The compositions of bridgmanite from starting materials A and B were Mg_0.91_Fe_0.10_Al_0.08_Si_0.91_O_3_ (lower Al/Fe ratio) and Mg_0.88_Fe_0.11_Al_0.13_Si_0.88_O_3_ (higher Al/Fe ratio), respectively, regardless of pressure.

The phases present in the recovered sample were identified through X-ray diffraction using a Brucker AXS D8 Discover micro-focused diffractometer at BGI. This was equipped with a two-dimensional solid-state detector and a Co-Kα radiation source operated at 40 kV and 500 μA. Diffraction patterns were acquired from an area of ~100 μm^2^ for 2 h.

Samples were then double polished to obtain ~80-μm thin sections. The Fe^3+^/ΣFe ratios were determined from Mössbauer spectra collected from these thin sections using a constant acceleration Mössbauer spectrometer operating in transmission mode with a nominal 370 MBq ^57^Co point source in a 12-μm Rh matrix at room temperature. The velocity scale was calibrated with a 25-μm-thick α-Fe foil. A Ta foil with a 200-μm diameter hole was used to select the collection area. Spectra were collected for 1‒3 days, then folded and fitted using the MossA programme^48^ in the full transmission integral mode using multiple pseudo-Voigt line shapes. Following the procedure in our previous studies^9,18^, we deconvoluted the spectra using three quadrupole doublets. The three quadrupole doublets are considered to be from Fe^3+^ and Fe^2+^ in bridgmanite and Fe^2+^ in ferropericlase. We ignored Fe^3+^ in ferropericlase and Fe^0^ in the Ir-Fe alloy as they have negligible contributions. The Mössbauer hyperfine parameters (Supplementary Table 2) obtained from these models were in good agreement with previous studies^9,18,49^.

### Determination of the oxygen fugacity

Oxygen fugacity was measured using an Ir-Fe alloy redox sensor employing the equilibrium^19^

3.1 $$2{\rm{Fe}}+{{\rm{O}}}_{2}=2{\rm{FeO}}$$

The oxygen fugacity at a specific pressure and temperature can be calculated from the standard-state Gibbs free energy of reaction (equation (3.1)) and the activities of the Fe component in the Ir-Fe alloy (${a_{\text{Fe}}}^{\text{alloy}}$) and FeO component in ferropericlase (${a_{\text{FeO}}}^{\text{Fp}}$) using, where *R* is the gas constant and *T* the temperature,

3.2 $$\log \left(\;{f}_{{\text{O}}_{2}}\right)=\frac{\Delta {{G}_{P,T,\mathrm{eq}3.1}}^{0}}{\mathrm{ln}\left(10\right)RT}+2\log \left({a_{\text{FeO}}}^{\text{Fp}}\right)-2\log \left({a_{\text{Fe}}}^{\text{alloy}}\right)$$

For fugacities reported relative to iron-wüstite (IW), the first term on the right-hand side of equation (3.2) becomes zero, and the relative oxygen fugacity is calculated as

3.3 $$\begin{array}{l}\Delta {IW}=2\log \left({a_{\text{FeO}}}^{\text{Fp}}\right)-2\log \left({a_{\text{Fe}}}^{\text{alloy}}\right)=2\log \left({\gamma _{\text{FeO}}}^{\text{Fp}}\times{x_{\text{FeO}}}^{\text{Fp}}\right)\\\qquad\quad-2\log \left({\gamma _{\text{Fe}}}^{\text{alloy}}\times{x_{\text{Fe}}}^{\text{alloy}}\right)\end{array}$$

${x_{\mathrm{Fe}}}^{\mathrm{alloy}}$ and ${\gamma_{\mathrm{Fe}}}^{\mathrm{alloy}}$ and ${x_{{\rm{FeO}}}}^{{\rm{Fp}}}$ and ${\gamma_{\mathrm{FeO}}}^{\mathrm{Fp}}$ are, respectively, the mole fraction and activity coefficient of the iron component in the Ir-Fe alloy and the FeO component in ferropericlase. The activity coefficient of Fe in Ir-Fe alloy was given by Stagno and Frost^19^. The symmetric Margules interaction parameter of FeO in ferropericlase was given by Frost^50^. The absolute oxygen fugacity used in the model calculations employed the value for IW calculated using data from Dorogokupets et al.^51^ for FCC iron and HCP iron and Stixrude and Lithgow–Bertelloni^32^ for FeO.

### Thermodynamics approach

For each endmember, the standard-state Gibbs free energy was calculated using the thermodynamic framework developed by Stixrude and Lithgow–Bertelloni^32,52^. The computations used the Burnman software^53,54^, which gave the standard-state Gibbs free energies of each endmember and the contributions due to ideal and non-ideal mixing. For bridgmanite, the model includes Mg^2+^, Fe^2+^, Fe^3+^ and Al^3+^ mixing on the A site and Al^3+^, Fe^3+^ and Si^4+^ mixing on the B site. In addition, oxygen vacancies can form on one half of the O1 sites^9^, so, equilibrium (2.1) is in fact

3.4 $${2\left[{\rm{Mg}}\right]}_{{\rm{A}}}{[{\rm{Al}}]}_{{\rm{B}}}[{{\rm{O}}}_{0.5}{{\rm{V}}}_{0.5}]{{\rm{O}}}_{2}\left({\rm{Bdm}}\right)={[{\rm{Al}}]}_{{\rm{A}}}{[{\rm{Al}}]}_{{\rm{B}}}{[{\rm{O}}]{\rm{O}}}_{2}({\rm{Bdm}})+2\left[{\rm{Mg}}\right]{\rm{O}}\left({\rm{Fp}}\right)$$

where V denotes an oxygen vacancy, and A and B refer to the respective sites in bridgmanite. On the basis of equilibrium (3.4) and the vacancy occupancy at O1, a normalization constant of 2 is required so that the ideal mixing activity of pure endmember MgAlO_2.5_ is equal to unity.

Thermodynamic parameters were initially taken from an existing database^32,52^ and our previous studies^9,18^. However, when the model used the parameters from these previous studies, there were some deviations from the data obtained in this study, especially for the concentration of the OV component. As a recent study determined that the volume of the MgAlO_2.5_ component is much smaller than its previous estimate^27^, we used this new volume for the OV component and redetermined the interaction parameters between it and the other components. In this optimization, we manually adjusted the interaction parameters due to the high correlation between the interaction parameters that a computer-automated optimization does not account for. This parameter optimization involved minimizing the sum of the squared partial Gibbs free energy changes over equations (2.1)–(2.4):

3.5 $${{{\chi }}}^{2}=\mathop{\sum }\limits_{i=1}^{\mathrm{reactions}}{\varDelta {{\rm{\mu }}}_{i}}^{2}$$

The resulting parameters are listed in Supplementary Tables 3‒5.

## Online content

Any methods, additional references, Nature Portfolio reporting summaries, source data, extended data, supplementary information, acknowledgements, peer review information; details of author contributions and competing interests; and statements of data and code availability are available at 10.1038/s41561-025-01725-0.

## Supplementary information

Supplementary Information Supplementary Figs. 1–7 and Tables 1–6.

## Data Availability

The data supporting the findings of this study are provided in the Supplementary Information. These data are also available via Zenodo at 10.5281/zenodo.15338042 (ref. ^55^).
