# Supplementary material for: Bridgmanite’s ferric iron content determined Earth’s oxidation state
Source: Nat Geosci. 2025 Jun 23;18(7):670–4. doi: 10.1038/s41561-025-01725-0 (PMC12245712; doi:10.1038/s41561-025-01725-0)
Supplement: Supplementary file 1 — Supplementary Figs. 1–7 and Tables 1–6. [file 41561_2025_1725_MOESM1_ESM.pdf]

nature geoscience

Supplementary information

https://doi.org/10.1038/s41561-025-01725-0

Bridgmanite's ferric iron content determined Earth's oxidation state

In the format provided by the authors and unedited

The PDF file includes:

Supplementary Figures 1 – 7

Supplementary Tables 1 – 6

Figure S1. Back-scattered electron images of the products from starting material A (Fe10Al8) showing texturally well equilibrated equant grains of bridgmanite (Bdm) coexisting with ferropericlase (Fp) and Ir-Fe alloy (the redox sensor).

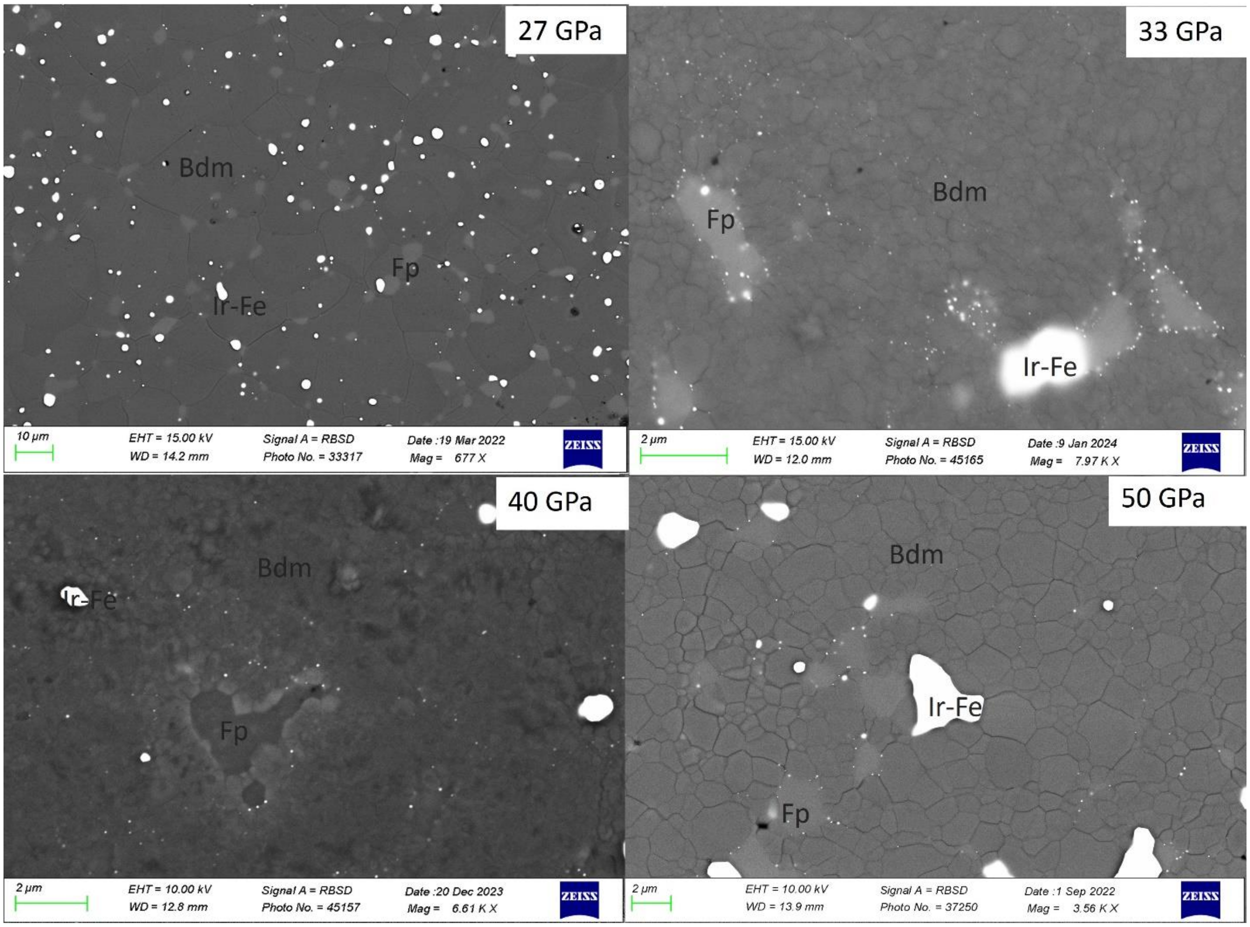

Figure S2. Back-scattered electron images of the products from starting material B (Fe11Al13) showing texturally well equilibrated equant grains of bridgmanite (Bdm) coexisting with ferropericlase (Fp) and Ir-Fe alloy (the redox sensor).

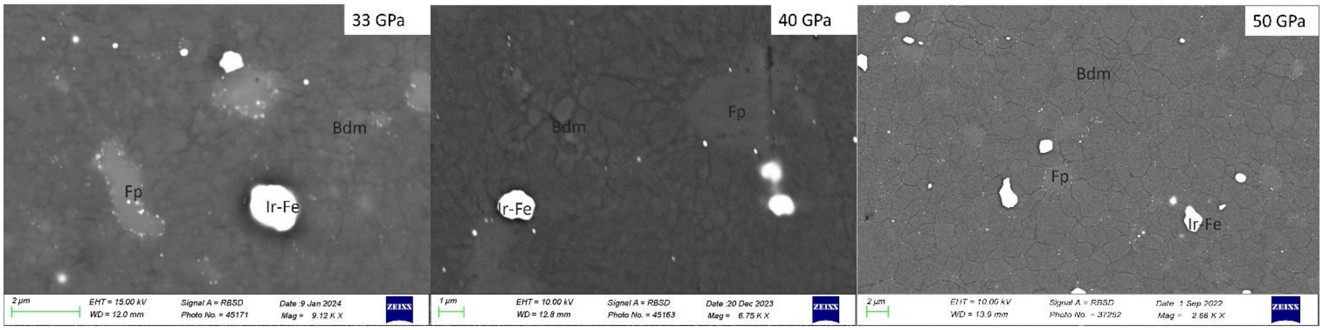

Figure S3. Representative room temperature Mössbauer spectra of run products at 50 GPa from the B (left) and A (right) bulk compositions. The doublets are Fe^{3+} in Bdm (dark blue), Fe^{2+} in Bdm (cyan) and Fe^{2+} in Fp (green). The experimental fit residuals are shown at the top

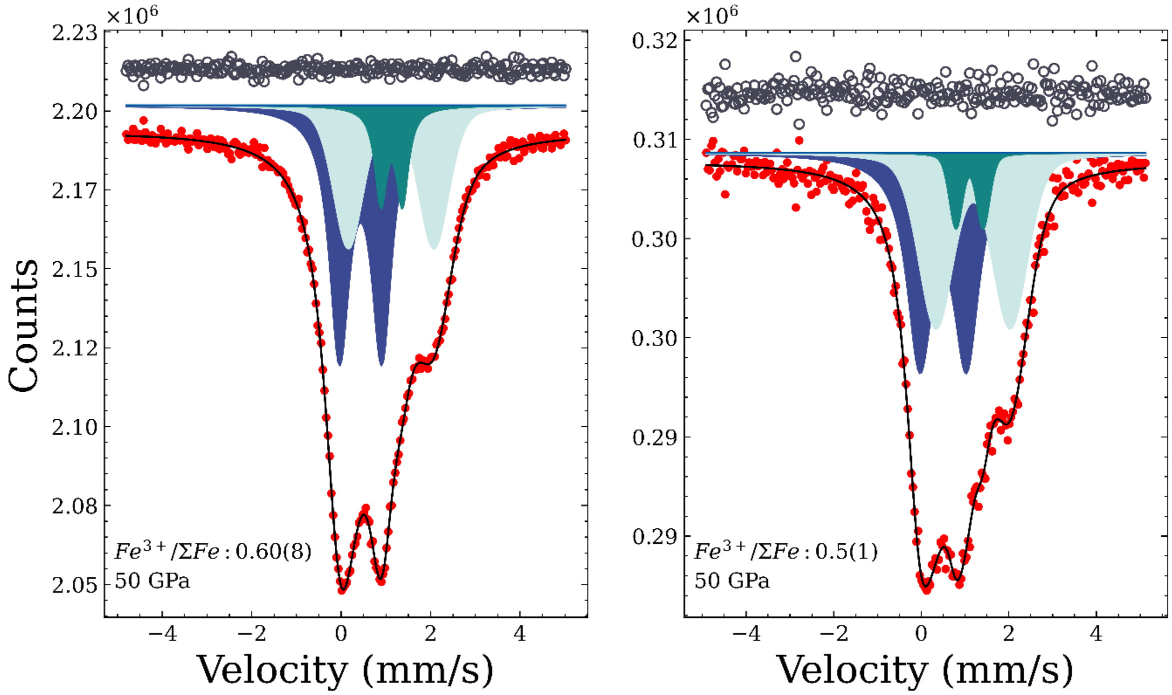

Figure S4. Comparison with literature bridgmanite $Fe^{3+}/\Sigma Fe$ ratios determined in experiments over wide ranges of pressure (27–110 GPa), temperature (1800–2600 K), Al/Fe ratio (0–1.3) and oxygen fugacity (IW -2 to +8). The solid diagonal line indicates 1:1 agreement with our thermodynamic model and the dashed lines show ±0.10 fluctuations from 1:1. Experimental data shown with solid symbols have the oxygen fugacity either controlled or measured. An open symbol indicates that the experiment did not report an oxygen fugacity for which we assume those experiments that reported iron metal in the sample cell have an oxygen fugacity of IW -1. Data denoted as from this work are experimental values ±1σ (standard deviation). Other data are the values and error bars reported in the cited references and are usually also experimental values ±1σ. Those that did not report iron metal are assumed to have an oxygen fugacity of IW, consistent with measurements of the f_{O2} of unbuffered experiments in a diamond anvil cell[55]. For both, we assume an uncertainty of f_{O2} of one log unit. Data are from Andrault et al.[10]: gray diamonds; Shim et al.[30]: wine-red circles; Boujibar et al.[31]: pink crosses, Piet et al.[29]: purple stars, Prescher et al.[28]: red pentagrams, Huang et al.[9]: blue circles, Ishii et al.[24]: green box, Wang et al.[18]: yellow reversed triangles and this study: black X-crosses.

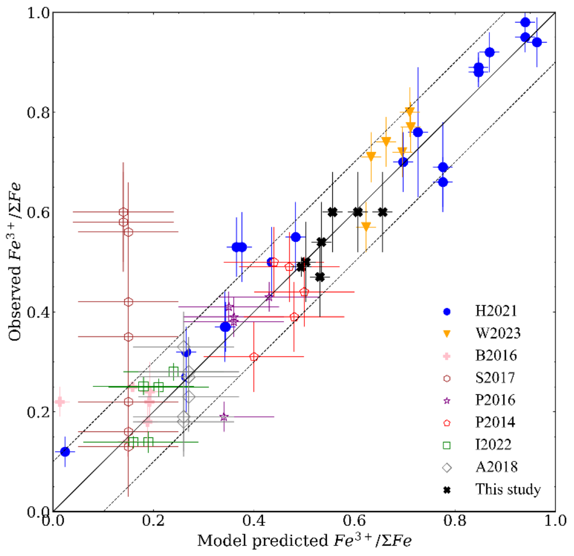

Figure S5. Proportion of $MgAlO_{2.5}$ bridgmanite oxygen vacancy and charge coupled components A) versus pressure, at 2000 K and a composition similar to those in Liu et al.[23]; B) versus temperature, at 27 GPa and a composition the same as in Liu et al.[21] and C) versus Al content, at 27 GPa and 2000 K. Data in sub-plot C are from Liu et al.[21,23,56]; Gruninger et al.[57]; Kojitani et al.[58] and Navrotsky et al.[59]. Data are the values and error bars reported in the cited references and are usually experimental values ±1σ. The curves were calculated using the equations referenced in the text, using compositional data calculated by the thermodynamic model using the thermodynamic parameters described in the text.

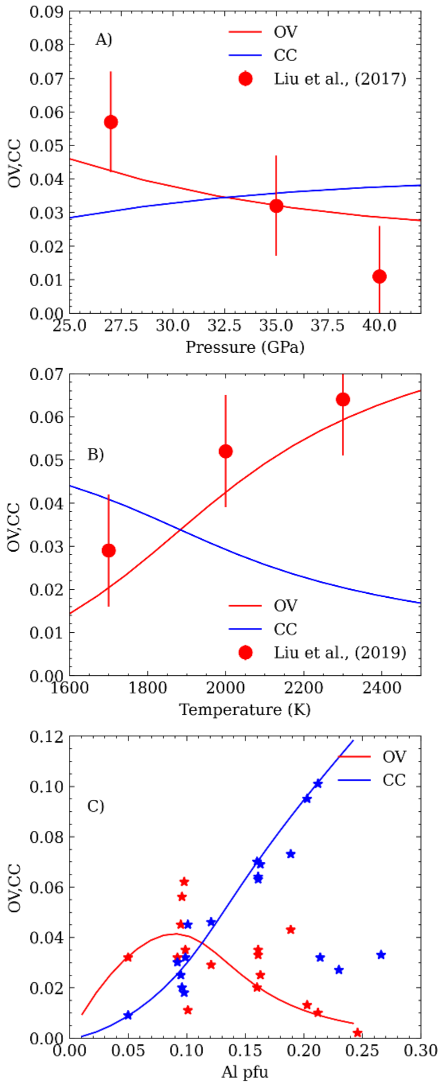

Figure S6. The early Earth's lower mantle solidus compared with the iron melting curve. Dashed and dotted lines are, respectively, mantle solidi and melting curves of iron in the literature (Andrault et al.,[34]; Anzellini et al.,[60]; Fiquet et al.,[35]; Hou et al.,[61]; Nomura et al.[33]; Pierru et al.,[36]; Zhang et al.,[62]). The red and green lines are averages, respectively, of the mantle solidi and iron melting curves. Although an individual mantle solidus and an iron melting curve are less coincident, the averaged solidus and melting curve show that within uncertainty, both temperature profiles are the same.

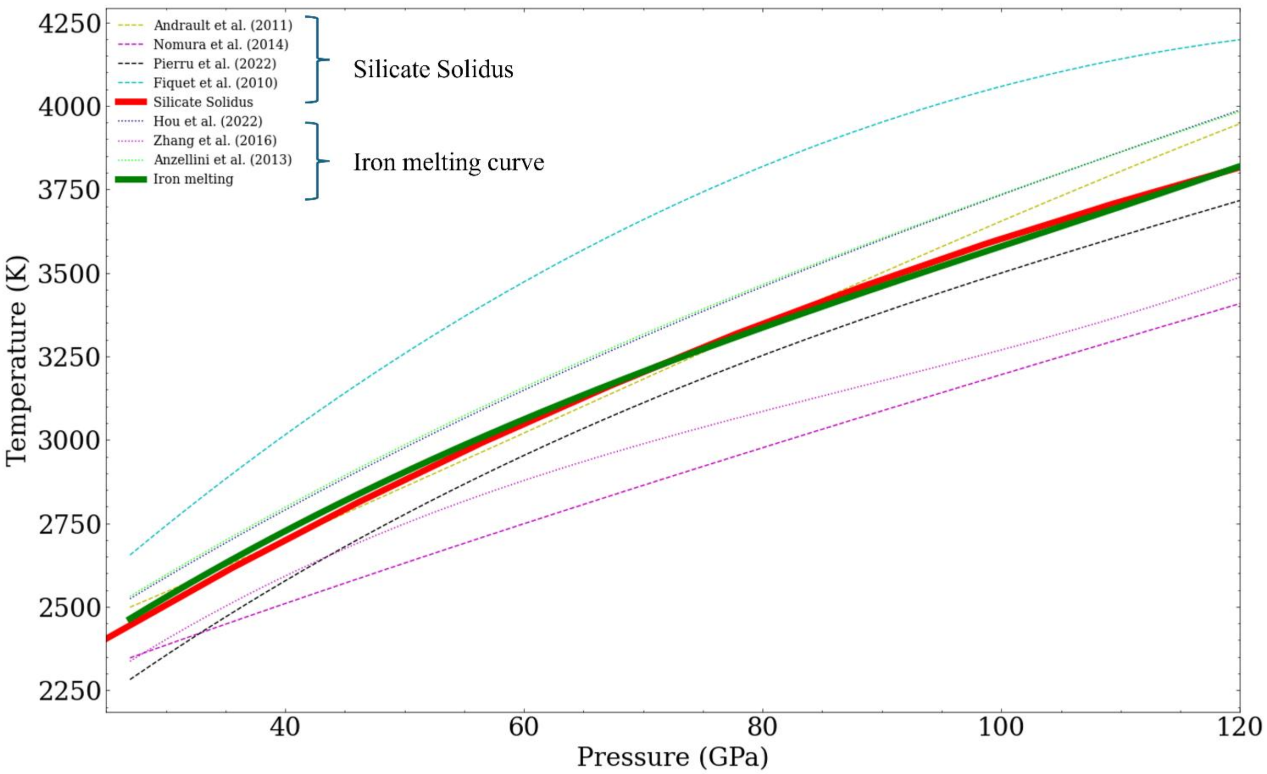

Figure S7. Cell assembly used in this study for > 27 GPa experiments.

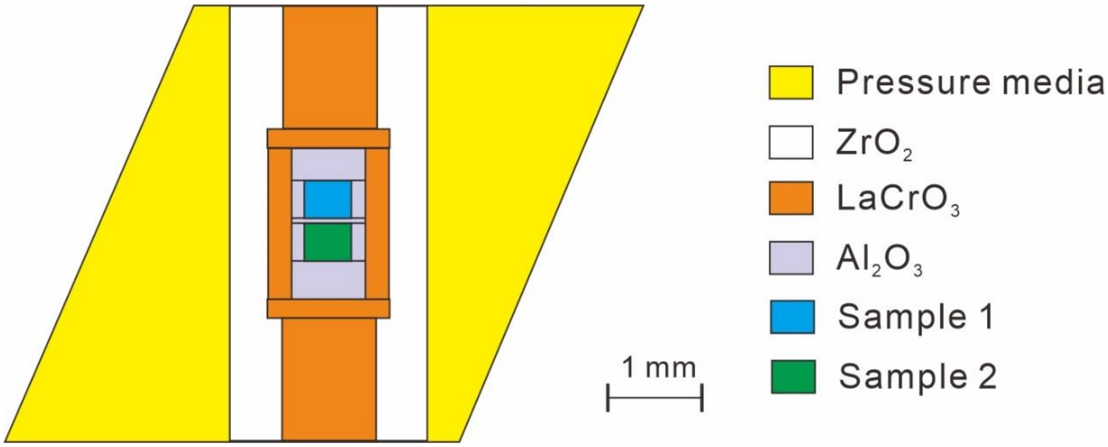

Table S1. Experimental conditions and composition in wt.% of Bdm, Fp and Ir-Fe and cation proportions normalized by number of atoms per formula unit in run products. All experiments were conducted at 2300 K.

			Oxide	SiO_2	MgO	FeO	Al_2O_3	IrO_2							
Run#	Phase	No.	metal	*Si*	*Mg*	*Fe*	*Al*	*Ir*	Total	Mg	Si	Fe	Al	Ir	ΣCat.
33GPa I1339 A13Fe11 log f_{O_2} (ΔIW):1.5(1)															
	Bdm	32		*34.3(8)*	*51.6(7)*	*7.6(2)*	*6.4(2)*	*0.3(3)*	*100.1(9)*	*0.88(2)*	*0.88(1)*	*0.109(3)*	*0.130(4)*	*0.001(1)*	*2*
	Fp	17		*78.3(6)*	*0.4(3)*	*21.5(4)*	*0.39(5)*	*0.4(3)*	*100.8(3)*	*0.861(7)*	*0.003(2)*	*0.132(2)*	*0.0034(4)*	*0.0007(6)*	*1*
	Ir-Fe	13		*0.2(2)*	*0.2(2)*	*4.5(5)*	*0.0(0)*	*93(2)*	*98(2)*	*0.01(1)*	*0.01(1)*	*0.14(2)*	*0.0*	*0.83(2)*	*1*
40GPa I1374 A13Fe11 log f_{O_2} (ΔIW):1.9(2)															
	Bdm	30		*34.3(3)*	*52.0(3)*	*8.0(3)*	*6.5(2)*	*0.2(3)*	*100.9(4)*	*0.870(7)*	*0.885(5)*	*0.114(4)*	*0.130(5)*	*0.001(1)*	*2*
	Fp	10		*86(1)*	*1(1)*	*13.0(7)*	*0.48(9)*	*0.3(2)*	*100.8(3)*	*0.91(1)*	*0.010(7)*	*0.077(4)*	*0.0040(8)*	*0.0005(4)*	*1*
	Ir-Fe	11		*0.2(3)*	*0.2(2)*	*1.6(4)*	*0.0(0)*	*97(1)*	*99(1)*	*0.01(2)*	*0.01(1)*	*0.05(1)*	*0.0*	*0.92(1)*	*1*
50GPa I1414 A13Fe11 log f_{O_2} (ΔIW):0.9(1)															
	Bdm	35		*34.4(3)*	*52.0(4)*	*7.7(4)*	*6.4(2)*	*0.3(3)*	*100.7(6)*	*0.874(8)*	*0.888(6)*	*0.109(5)*	*0.128(4)*	*0.001(1)*	*2*
	Fp	11		*82.0(6)*	*0.8(4)*	*17.4(4)*	*0.58(7)*	*0.1(2)*	*100.9(4)*	*0.884(6)*	*0.006(3)*	*0.105(2)*	*0.0049(6)*	*0.0003(4)*	*1*
	Ir-Fe	10		*0.6(3)*	*0.7(4)*	*4.7(6)*	*0.003(7)*	*93(1)*	*99(1)*	*0.04(2)*	*0.04(2)*	*0.14(2)*	*0.0002(4)*	*0.78(1)*	*1*
27GPa I1319 A7Fe10 log f_{O_2} (ΔIW):1.3(1)															
	Bdm	38		35.2(3)	53.5(5)	6.6(3)	4.0(2)	0.3(2)	99.6(6)	0.903(8)	0.919(8)	0.095(5)	0.082(3)	0.001(1)	2
	Fp	20		80(1)	0.11(9)	19.4(8)	0.80(6)	0.3(2)	101.0(7)	0.87(1)	0.001(1)	0.118(5)	0.007(1)	0.0005(5)	1
	Ir-Fe	18		0.03(3)	0.04(4)	5.4(5)	0.0(0)	95(1)	100(1)	0.002(2)	0.002(3)	0.16(2)	0.0	0.833(9)	1
33GPa I1339 A7Fe10 log f_{O_2} (ΔIW):1.7(1)															
	Bdm	20		35.6(2)	53.8(4)	6.9(2)	4.0(1)	0.5(2)	100.8(5)	0.905(4)	0.916(7)	0.099(3)	0.080(3)	0.002(1)	2
	Fp	18		78.8(8)	0.5(5)	21.1(7)	0.23(5)	0.3(3)	100.9(5)	0.864(9)	0.004(3)	0.130(4)	0.0020(4)	0.0006(6)	1
	Ir-Fe	13		0.09(6)	0.1(1)	3.6(5)	0.0(0)	95(1)	99(1)	0.006(4)	0.008(8)	0.11(2)	0.0	0.87(1)	1
40GPa I1374 A7Fe10 log f_{O_2} (ΔIW):1.6(3)															
	Bdm	15		35.9(3)	53.6(3)	7.3(3)	4.0(1)	0	101.1(5)	0.907(8)	0.910(5)	0.103(4)	0.081(2)	0.0	2
	Fp	15		84.5(8)	0.5(6)	15.4(5)	0.27(5)	0.3(2)	101.0(5)	0.901(9)	0.004(5)	0.092(3)	0.0022(5)	0.0005(4)	1
	Ir-Fe	12		0.2(2)	0.3(3)	2.5(8)	0.0(0)	97(1)	100(1)	0.01(1)	0.02(2)	0.08(3)	0.0	0.89(1)	1
50GPa I1414 A7Fe10 log f_{O_2} (ΔIW):1.2(1)															
	Bdm	34		35.5(3)	54.2(5)	6.9(4)	4.0(1)	0.3(3)	100.9(4)	0.900(8)	0.920(9)	0.098(6)	0.080(2)	0.001(1)	2
	Fp	17		83(1)	0.6(6)	16.8(8)	0.43(5)	0.3(3)	100.9(7)	0.89(1)	0.004(5)	0.102(5)	0.0037(4)	0.0006(5)	1
	Ir-Fe	14		0.4(3)	0.2(2)	3.2(5)	0.0(0)	95(1)	99(1)	0.03(2)	0.010(9)	0.10(2)	0.0	0.863(9)	1

Table S2. Best-fit hyperfine parameters of the Mössbauer spectra and the $Fe^{3+}/\Sigma Fe$ ratios in bridgmanite.

P(GPa)	Sample	Fe^{2+}			Fe^{3+}			$Fe^{3+}/\Sigma Fe$ (Bdm)	Fe^{2+}(Fp)		
		CS[a]	QS[b]	Areas	CS[a]	QS[b]	Areas		CS[a]	QS[b]	Areas
50	Al13Fe11	1.117(47)	1.92(15)	35.5(6.8)	0.436(45)	0.95(13)	52.5(7.3)	60(8)	1.129(83)	0.48(22)	12.1(9.2)
50	Al7Fe10	1.18(11)	1.70(40)	44(16)	0.500(66)	1.07(11)	49(17)	50(10)	1.102(59)	0.621(95)	7(26)
40	Al13Fe11	0.975(54)	2.09(16)	32(15)	0.48(13)	0.76(15)	43(14)	57(8)	1.02(21)	0.53(42)	25(20)
40	Al7Fe10	1.054(31)	1.74(11)	44(11)	0.330(22)	0.840(41)	42.4(9.1)	49(8)	1.109(57)	0.382(49)	14(13)
33	Al13Fe11	0.95(11)	2.04(20)	28(17)	0.49(15)	0.767(64)	49(16)	64(8)	1.06(27)	0.69(45)	22(19)
33	Al7Fe10	1.130(23)	1.83(13)	44.6(9.4)	0.431(22)	1.077(39)	40.0(8.0)	47(7)	1.073(22)	0.585(92)	15(13)
27	Al7Fe10	1.136(10)	1.729(43)	47.4(2.6)	0.4660(80)	1.001(17)	45.2(2.4)	49(2)	1.0830(70)	0.545(26)	7.4(3.8)

[a] Relative to α-Fe unit: mm/s

[b] unit: mm/s

Table S3. Parameters from the literature and this study for the Gibbs free energy of end members of Bdm and Fp.

	F_0 (kJ/mol)	V_0 (cc/mol)	K (GPa)	K′	Θ_0 (K)	γ_0	q_0
$MgSiO_3$ (Bdm)	-1362.443[c]	24.45[c]	251[c]	4.1[c]	880[c]	1.54[c]	0.84[c]
$FeSiO_3$ (Bdm)	-1002.663[c]	25.32[c]	271[c]	4.01[c]	746[c]	1.54[c]	0.84[c]
$FeFeO_3$ (Bdm)	-537.030[d]	29.55[d]	157[d]	4[d]	444[d]	1.54[d]	0.84[d]
$AlAlO_3$ (Bdm)	-1517.729[c]	24.94[c]	242[c]	4.1[c]	858[c]	1.54[c]	0.84[c]
$FeAlO_3$ (Bdm)	-1107.692[d]	27.02[d]	220[c]	4.1[d]	697[d]	1.54[d]	0.84[d]
$MgAlO_{2.5}$ (Bdm)	-1295.600[a]	24.39[a]	155[a]	4.1[a]	749[a]	1.54[a]	0.84[a]
Mg_4O_4 (Fp)	-2278.110[c]	44.97[c]	161[c]	3.9[c]	771[c]	1.45[c]	1.55[c]
Fe_4O_4 (Fp)	-974.607[c]	49.02[c]	179[a]	4[c]	454[c]	1.45[c]	1.55[c]
Fe_4O_4(Fp ls)	-609.335[b]	43.39[b]	200[b]	4.0[b]	492[b]	1.549[b]	1.55[b]

[a]This study. [b]Criniti et al.[27]. [c]Stixrude & Lithgow Bertelloni [32]. [d]Wang et al. [18]

Table S4. Margules interaction parameters for non-ideal mixing in bridgmanite solution.

W_{ij} (KJ/mol)	$MgSiO_3$	$FeSiO_3$	$FeFeO_3$	$AlAlO_3$	$FeAlO_3$	$MgAlO_{2.5}$
$MgSiO_3$		-11.7[a]	0	35 [a]	5	-4
$FeSiO_3$		0	0	0	0	0
$FeFeO_3$			0	0	0	0
$AlAlO_3$				0	0	230
$FeAlO_3$					0	-100

[a]from Stixrude & Lithgow Bertelloni [32]. The other parameters are from this study

Table S5. Margules interaction parameters for non-ideal mixing in ferropericlase solution.

W_{ij} (KJ/mol)	MgO	FeO
MgO		13.7[a]

[a]from Frost [50]

Table S6. Starting material with 95.5% 57Fe isotope enrichment.

Series	MgO (wt%)	SiO_2 (wt%)	Al_2O_3 (wt%)	Fe_2O_3 (wt%)
A	35.3	52.1	3.9	8.7
B	34.2	50.6	6.0	9.2

Reference

55. Dominijanni, S. Physicochemical properties of Fe-bearing minerals and metal alloys at deep Earth conditions. (University of Bayreuth, Bayreuth, 2022).

56. Liu, Z., Boffa Ballaran, T., Huang, R., Frost, D. J. & Katsura, T. Strong correlation of oxygen vacancies in bridgmanite with Mg/Si ratio. *Earth Planet. Sci. Lett.* **523**, 115697 (2019).

57. Grüninger, H. *et al.* Oxygen Vacancy Ordering in Aluminous Bridgmanite in the Earth's Lower Mantle. *Geophys. Res. Lett.* **46**, 8731–8740 (2019).

58. Kojitani, H., Katsura, T. & Akaogi, M. Aluminum substitution mechanisms in perovskite-type $MgSiO_3$: an investigation by Rietveld analysis. *Phys. Chem. Miner.* **34**, 257–267 (2007).

59. Navrotsky, A. *et al.* Aluminum in magnesium silicate perovskite: Formation, structure, and energetics of magnesium-rich defect solid solutions. *J. Geophys. Res. Solid Earth* **108**, 2002JB002055 (2003).

60. Anzellini, S., Dewaele, A., Mezouar, M., Loubeyre, P. & Morard, G. Melting of Iron at Earth's Inner Core Boundary Based on Fast X-ray Diffraction. *Science* **340**, 464–466 (2013).

61. Hou, M. *et al.* Melting of Iron Explored by Electrical Resistance Jump up to 135 GPa. *Geophys. Res. Lett.* **48**, e2021GL095739 (2021).

62. Zhang, D. *et al.* Temperature of Earth's core constrained from melting of Fe and $Fe_{0.9}Ni_{0.1}$ at high pressures. *Earth Planet. Sci. Lett.* **447**, 72–83 (2016).
